# Neurosarcoidosis: a clinical approach to diagnosis and management

**DOI:** 10.1007/s00415-016-8336-4

**Published:** 2016-11-22

**Authors:** Richard T. Ibitoye, A. Wilkins, N. J. Scolding

**Affiliations:** 1 Department of Neurology, Southmead Hospital, Southmead Road, Bristol, BS10 5NB UK; 2 Institute of Clinical Neurosciences, University of Bristol, Bristol, BS10 5NB UK

**Keywords:** Sarcoidosis, Neurosarcoidosis, Diagnosis, Treatment, Management

## Abstract

Sarcoidosis is a rare but important cause of neurological morbidity, and neurological symptoms often herald the diagnosis. Our understanding of neurosarcoidosis has evolved from early descriptions of a uveoparotid fever to include presentations involving every part of the neural axis. The diagnosis should be suspected in patients with sarcoidosis who develop new neurological symptoms, those presenting with syndromes highly suggestive of neurosarcoidosis, or neuro-inflammatory disease where more common causes have been excluded. Investigation should look for evidence of neuro-inflammation, best achieved by contrast-enhanced brain magnetic resonance imaging and cerebrospinal fluid analysis. Evidence of sarcoidosis outside the nervous system should be sought in search of tissue for biopsy. Skin lesions should be identified and biopsies taken. Chest radiography including high-resolution computed tomography is often informative. In difficult cases, fluorodeoxyglucose positron emission tomography and gallium-67 imaging may identify subclinical disease and a target for biopsy. Symptomatic patients should be treated with corticosteroids, and if clinically indicated other immunosuppressants such as hydroxychloroquine, azathioprine, cyclophosphamide or methotrexate should be added. Anti-tumour necrosis factor alpha therapies may be considered in refractory disease but caution should be exercised as there is evidence to suggest they may unmask disease.

## Introduction

### Purpose of review

Neurosarcoidosis (NS) is a rare neuro-inflammatory disorder with protean manifestations which presents a diagnostic challenge to general physicians and neurologists alike. In 2007, we highlighted how case series continued to refine our understanding, and that anti-tumour necrosis factor alpha (TNF-α) therapies may hold promise as steroid-sparing agents in difficult disease [1, 2]. Nine years on, we provide a clinically focused update on the management of NS, guided by knowledge from new case series, refinements in diagnostic criteria and further reports on anti-TNF-α and other immunosuppressive therapies.

### Definition and epidemiology

Sarcoidosis is a multi-organ granulomatous disease of unknown aetiology, and is characterised pathologically by multiple non-caseating granulomata in the absence of a defined infective or toxic trigger [3]. In a US based study, incidence (in person-years) was estimated at 10.9/100,000 in Americans of European ancestry and 35.5/100,000 in those of African ancestry [4].

Neurosarcoidosis, the involvement of the nervous system by sarcoid granulomata, is uncommon, occurring symptomatically in 5–16% of patients with sarcoidosis [5–8]. Our regional hospital-based study of patients in South West England and South Wales estimated a prevalence of 1/100,000 [9]. Across two case series, we found a mean age at diagnosis of 39 years and similar prevalence in females and males (47 vs. 53% across 98 patients) [2, 9]. A significant proportion of NS is subclinical as confirmed by autopsy studies; one showed nervous system involvement in 14% of those with sarcoidosis [10]; another found only in 50% was NS diagnosed in life [11].

## Background

The first case description of sarcoidosis is attributed to Hutchinson in 1869 when he described a coal-wharf worker with skin changes in his hands and legs [12]. The first account of a neurological syndrome attributable to sarcoidosis was by Heerfordt, who in 1909 reported three males with uveitis, parotid enlargement and fever; two had a facial nerve palsy [13]. Kveim’s observation that sarcoid lymph node tissue generated an immunological response when injected intradermally in patients with sarcoidosis, provided evidence for a non-tubercular basis to the uveoparotid syndrome [14]. Siltzbach and colleagues subjected this to clinical trial and confirmed the Kveim–Siltzbach test highly specific (95%) and sensitive (79%) for sarcoidosis [15].

Case series with heterogeneous definitions of NS expanded our understanding of NS beyond Heerfordt’s uveoparotid fever [16]. Zajicek et al.’s diagnostic criteria provided a much needed framework for case definition, separating definite disease (with a positive biopsy from the nervous system) from probable and possible disease [2]. Refinements of these criteria have since been proposed, guided by emerging technologies such as thoracic high-resolution computed tomography (HRCT), and immunohistochemistry for CD4:CD8 lymphocyte ratios in broncho-alveolar lavage (BAL) specimen [17].

### Natural history

The natural history of NS is poorly characterised due to its rarity, variability in diagnostic criteria and the frequent use of corticosteroid or immunosuppressive therapy. Large case series show neurological symptoms and signs herald the diagnosis of sarcoidosis in 31–71% [2, 6, 9, 18–20]. In those with established sarcoidosis, NS can develop years after disease onset [6, 9, 18].

NS may be monophasic, relapsing or chronic and outcomes relate to disease phenotype. Intracranial or spinal mass lesions often relapse on corticosteroids following dose reduction [9]. Optic neuritis tends also to have a relapsing phenotype [2]. Facial mononeuropathies on the other hand often remit, carrying a better prognosis [6, 21]. Myopathy and peripheral neuropathy are less common and can be chronic [22].

## Diagnosis

### When should neurosarcoidosis be suspected?

From a Bayesian perspective, there are two clinical scenarios in which it would be reasonable to suspect NS. These are: (1) the development of a neurological problem in a patient known to have sarcoidosis and (2) a patient presenting with a neurological syndrome ‘typical’ for NS. Patients with cryptogenic neuro-inflammatory disease in whom common mimics have been excluded should also be investigated for NS.

In the patient with systemic sarcoidosis who develops new neurological symptoms and signs, the likelihood of any such neurological presentation being due to sarcoidosis is high—with the proviso that individuals who have received immunosuppression as part of their previous sarcoid treatment may be at greater risk of CNS infections, which, therefore, must be rigorously excluded. The likelihood becomes higher still once the problem is characterised as being neuro-inflammatory. Such reasoning is captured in Zajicek’s criteria for probable NS [2].

Similarly, should any neurological syndrome be uncommon in general, but common in NS, its emergence would support a search for sarcoidosis as the basis for disease. A wide range of presentations are reported, but of these: the uveoparotid syndrome and cranial oligoneuropathy (for example a bilateral facial neuropathy) most readily meet these conditions (see Table 1), while long lesions of the cervical or thoracic spine, a cauda equina syndrome, or pituitary and/or hypothalamic involvement, with or without obstructive hydrocephalus, are also characteristic of CNS sarcoidosis.

**Table 1:** Clinical presentations and their characteristics as reported in NS

Clinical presentation	Characteristics in NS
Aseptic meningitis [2, 6, 9, 18–22, 24]	Mostly this is asymptomatic and inferred from CSF abnormalities. Where patients are symptomatic, the presentation is usually subacute or chronic
	Typical CSF finds are: pleocytosis (<220 cells/mm^3^) with a lymphocytic predominance and/or raised protein (<4.3 g/l). Reduced CSF glucose is also reported
Conus or cauda equina syndrome [9, 19, 43]	This may be of acute or subacute onset. CSF and imaging abnormalities usually confirm a neuro-inflammatory basis
Cranial neuropathy [2, 6, 8, 18–22, 24]	This is the most frequently reported manifestation of NS. Any cranial nerve can be involved but facial and optic nerves are most frequently affected
	Facial nerve palsies often spontaneously remit and carry a good prognosis
	Cranial oligoneuropathy or polyneuropathy (e.g. bilateral facial nerve palsy) is suggestive of NS
	Optic nerve involvement may have a more difficult disease course with refractory disease and relapse on corticosteroid dose reduction
	A pharynx, soft palate and vocal cord syndrome from glossopharyngeal and vagus nerve involvement is recognised
	Basal meningitis may be the pathophysiological substrate of cranial neuropathies
Focal neurology, multifocal neurology or diffuse encephalopathy due to parenchymal lesions of the brain or brainstem [2, 8, 9, 18–22, 44]	Lesions may be multiple and often enhance. Biopsy of mass lesions is recommended for a definitive diagnosis
	Behaviour change, confusional states and psychosis are reported
Hypothamic and pituitary dysfunction [2, 6, 8, 9, 20, 22, 28, 29, 44–46]	Usually of insidious onset, due to suprasellar inflammatory lesions. The most eminent symptoms are bitemporal visual failure, polydipsia and polyuria (diabetes insipidus), and galactorrhoea
	Symptoms may arise from hypothalamic dysfunction, hypopituitarism or compression of the optic chiasm by mass effect
	An aseptic meningitis is often seen
Myopathy [6, 19, 20, 22, 38]	Usually asymptomatic. Where symptomatic, this presents as proximal weakness. Biopsy is reported to have a high diagnostic yield
Peripheral polyneuropathy [6, 8, 19, 20, 22, 24]	Pure sensory and mixed neuropathies are reported. Mononeuritis multiplex is also described
Raised intracranial pressure [2, 6, 9, 19–22]	Patients usually present non-specifically with a headache and visual disturbance. Clinical signs may include papilloedema
	CSF and imaging show evidence of active inflammation, including meningeal enhancement and ventriculitis
	Hydrocephalus may develop and may require surgical management
Seizures [8, 9, 18, 21, 22, 38]	Can be a feature of cortical or subcortical disease
Spinal cord syndromes and radiculitis [2, 6, 9, 20, 22–24]	Mass lesions and inflammatory lesions are reported. A Guillain–Barré-like syndrome is occasionally described
	In longitudinally extensive myelitis where aquaporin antibodies are negative, NS should be considered
Uveoparotid fever [13]	Uveitis, parotid gland swelling, fever and facial nerve palsy constitute this syndrome which is pathognomonic of sarcoidosis
	CSF often shows evidence of an aseptic meningitis
Vascular syndromes [8, 20, 47–49]	Ischaemic stroke, haemorrhagic stroke and dural venous sinus thrombosis are infrequently reported
	Perivascular inflammation has been demonstrated in biopsy and post-mortem specimen

Cryptogenic neuro-inflammatory disease is a not infrequent problem for the practising neurologist. In the authors’ view, following the exclusion of common infective and auto-immune causes, NS should be considered. Longitudinally extensive myelitis where serology for neuromyelitis optica is negative, is now recognised to often be secondary to NS [23].

### How should the diagnosis of neurosarcoidosis be established?

A definite diagnosis, on the basis of nervous system biopsy is preferred, but is not always practicable depending on the site of disease [2]. The diagnostic process for probable NS should otherwise involve confirming a neuro-inflammatory basis to disease and investigating for sarcoidosis.

### Confirming a neuro-inflammatory basis to disease

Enhancement on contrast-enhanced magnetic resonance imaging (MRI), as evidence of break-down of the blood–brain barrier, is a highly sensitive marker of neuro-inflammatory disease and is recommended in the investigation of NS. Where this is not practicable, contrast-enhanced computed tomography (CT) is an alternative. Cerebrospinal fluid (CSF) examination is helpful as constituents are often abnormal, with lymphocytic pleocytosis (in 31–83%) and elevated protein (in 40–83%) being typical; low glucose is occasionally identified, and an unmatched oligoclonal band pattern is often found (in 27–37%) [2, 6, 9, 18, 19, 24].

Symptomatic muscle involvement should lead to targeted biopsy, though success has been reported with blind biopsy in asymptomatic patients [25]. Imaging is less likely to demonstrate involvement of peripheral nerves, but electrophysiology may provide supporting evidence.

Where tissue is amenable to biopsy and the risk–benefit balance is favourable, nervous system tissue biopsy is recommended to confirm the diagnosis.

### Investigating for sarcoidosis

Sarcoidosis should be diagnosed on the basis of non-caseating granulomata in the absence of other granulomatous disease. There is a predilection for intrathoracic, skin and ocular tissues which guides investigation [26]. Chest radiography is often abnormal (in 31–82%), typically showing bihilar lymphadenopathy [2, 6, 9, 18, 20, 21, 23, 24, 27–29]. HRCT is more sensitive, and may identify areas for BAL or endobronchial ultrasound (EBUS)-guided biopsy [3, 30]. A BAL CD4:CD8 lymphocyte ratio of >3.5:1 is a well-validated marker, highly specific for sarcoidosis [31, 32]. Mediastinoscopy may permit biopsy of involved intrathoracic lymph nodes not accessible by EBUS. Where skin disease is present, dermatological review and biopsy is recommended. Ophthalmological assessment, though helpful in confirming ocular involvement, is less helpful for identifying tissue to biopsy.

Whole body fluorodeoxyglucose positron emission tomography (FDG-PET) and gallium-67 imaging are helpful investigations which identify asymptomatic involved tissue, may confirm a typical disease pattern and suggest a site for biopsy [33].

The Kveim–Siltzbach test has fallen into disuse. Safety concerns with regard transmissible infectious disease and exhaustion of existing antigen underlie this decline. Other investigations such as CSF or serum angiotensin converting enzyme, though often suggested as part of the work-up of NS, are insufficiently specific to be of diagnostic value [34, 35].

## Treatment

### What treatment should be initiated?

The natural history of NS is poorly defined, but spontaneous remission is recognised. Response to corticosteroids is the norm, but long-term outcomes are variable. Most are established on corticosteroids (e.g. prednisolone at 0.5–1 mg/kg) and 40–82% show sustained improvement or stability [2, 6, 18, 20, 29]. Case-mix differences most likely underlie differences in outcomes. In patients with a partial, or non-sustained response to steroids, or in whom long-term therapy is required, the usual practise is to establish a second-line immunosuppressant such as hydroxychloroquine, azathioprine, cyclophosphamide or methotrexate [1]. The successful use of cranial irradiation has been reported for difficult disease [36].

As macrophage-derived TNF-α plays a critical role in granuloma formation, anti-TNF-α therapies are hypothesised to be efficacious in NS. Successful outcomes in corticosteroid-refractory NS have been reported for infliximab (with 34 such reports as of 2014 [37]), and with adalimumab [38, 39]. Notwithstanding the risk of bias in retrospective case reports, the temporal correlation between administration and improvement, and the occurrence of sustained remission support a therapeutic effect [37, 40]. We did not find any reports of etanercept as a successful therapy in NS. There are sporadic reports of the use of other monoclonal biologic therapies in treatment-refractory NS.

A recently emerging potential complication of anti-TNF-α therapies has been the occurrence of new onset sarcoidosis-like disease in patients receiving these agents for other indications. As of 2012, 37 such cases had been described with 22 (60%) attributable to etanercept, suggesting an asymmetric class effect. Though paradoxical and unexpected, the close temporal association of sarcoidosis with anti-TNF-α therapies, resolution on cessation, and re-manifestation on repeat challenge support an aetiological role [41]—whilst challenging the previously accepted but arguably simplistic mechanism of action in treating sarcoidosis of blocking TNF-α.

### How should disease be monitored?

No reliable biomarkers have yet been identified to monitor disease activity in NS. The authors would advocate an individualised approach, guided by the neurological system involved. Visual acuity and colour vision testing may for example guide the monitoring of optic neuritis and contrast-enhanced MRI may support the monitoring of inflammatory lesions. PET-CT may play a role [42].

## Conclusion

Case series continue to broaden the phenotype of NS, reinforcing the need for a systematic approach to diagnosis and management. Adverse findings with anti-TNF-α therapies suggest they should be used with caution in NS.
